# ITULAZAX^®^ versus Alutard SQ^®^ in the treatment of allergic rhinitis induced by pollen from the birch homologous group: A cost‐minimization modeling analysis from the Danish societal perspective

**DOI:** 10.1002/clt2.12196

**Published:** 2022-11-03

**Authors:** Steen M. Rønborg, Tobias Sydendal Grand, Henrik Brandi, Richard F. Pollock

**Affiliations:** ^1^ Allergy and Pulmonology Clinic Vanløse Denmark; ^2^ ALK‐Abelló A/S Hørsholm Denmark; ^3^ Covalence Research Ltd Harpenden UK

**Keywords:** administration, allergic, costs and cost analysis, Denmark, desensitization, immunologic, oral, rhinitis

## Abstract

**Background and aims:**

Allergic rhinitis (AR) is an inflammatory disorder triggered by an allergic immune response to inhaled allergens. Birch pollen is the major allergenic tree pollen in parts of Europe. ITULAZAX® is a sublingual immunotherapy tablet for the treatment of adults with moderate‐to‐severe AR and/or conjunctivitis induced by pollen from the birch homologous group. The aim was to compare the costs of treating AR with ITULAZAX® versus subcutaneous ALUTARD SQ® *Betula verrucosa* (ALUTARD SQ®) from a Danish societal perspective.

**Methods:**

A cost‐minimization model was developed to capture costs of allergy immunotherapy (AIT), interactions with healthcare professionals (HCPs) in three different care settings (general practice, allergy specialist, and hospital), and indirect costs arising from absenteeism and presenteeism. The cost‐minimization analysis was conducted over a 3‐year time horizon with costs reported in 2021 Danish Kroner (DKK) and Euros (EUR) based on the European Central Bank 365‐day average exchange rate. One‐way sensitivity analyses were performed.

**Results:**

The base case analysis showed that the total cost of treatment over 3 years was estimated to be DKK 49,117 (EUR 6598) per patient with ALUTARD SQ®, compared with DKK 30,996 (EUR 4164) with ITULAZAX®, reflecting a cost saving of DKK 18,121 (EUR 2434) per patient with ITULAZAX® over 3 years. Over the 3‐year time horizon, costs of AIT were predicted to increase by DKK 17,928 (EUR 2408) with ITULAZAX®, while costs of interactions with HCPs were predicted to decrease by DKK 22,528 (EUR 3027) versus ALUTARD SQ®, more than offsetting the increased cost of ITULAZAX®.

**Conclusions:**

Given the equivalent effectiveness of the two AIT products, and the cost savings with ITULAZAX® versus ALUTARD SQ® from a Danish societal perspective, ITULAZAX® should be considered as a cost‐saving alternative to ALUTARD SQ® for the treatment of birch pollen‐induced moderate‐to‐severe AR in adults.

## 1 BACKGROUND AND AIMS

Allergic rhinitis (AR) is an inflammatory disorder triggered by an allergic immune response to inhaled allergens in individuals who have become sensitized to that particular allergen.^1^ Localized symptoms that subsequently arise from the immune cascade in the nasal mucosa can include nasal congestion or obstruction, rhinorrhea, an itchy nose, sneezing, and conjunctivitis, the last of which may manifest as ocular symptoms such as itchy or watery eyes, hyperemia, chemosis, and periorbital edema.^1^ In addition to these localized symptoms, systemic symptoms such as fatigue, reduced productivity, and impaired concentration are also commonly associated with AR.^1^ Furthermore, AR shares elements of pathology and pathophysiology with allergic asthma, which frequently co‐exists with AR in the same individual^2^, ^3^; up to 30% of patients with AR have concomitant asthma, and more than 70% of patients with asthma have concomitant AR.^4^, ^5^ AR and allergic asthma share a common systemic immunoglobulin E (IgE)‐mediated immunological response to inhaled allergens,^2^, ^6^ and AR has been identified as a key risk factor for developing asthma.^7^, ^8^, ^9^

The most common inhalant allergens causing AR include pollen, dust mites, dander, and insects.^1^, ^10^ Birch pollen is the major allergenic tree pollen in parts of Europe, and is also listed among the key pollen allergens in North America.^10^, ^11^ Birch pollen contains the major allergen Bet v 1, which is homologous with allergens from other trees in the *Fagales* order. The birch homologous group is defined by the European Medicines Agency as including alder, beech, hazel, hop/hornbeam, oak, and chestnut.^12^, ^13^ The cross‐reactive nature of allergens and the sequential flowering of trees in the birch homologous group can result in individuals with birch pollen‐induced AR experiencing symptoms for a prolonged period, extending beyond the birch pollen season.^14^ Cross‐reactivity may also expand the geographical area in which an allergic reaction may be triggered.

The diverse range of moderate‐severe symptoms combined with the long duration of birch and cross‐reactive allergen exposure can combine to make birch pollen‐induced AR a significant, debilitating disease that can affect many aspects of a person's life. Based on current guidelines, patients are encouraged to attempt to reduce symptoms of AR through allergen avoidance or, where allergen avoidance is neither possible nor effective, to take one of two forms of AR treatment recommended by clinical guidelines: allergy pharmacotherapy, or allergy immunotherapy (AIT). The Allergic Rhinitis and its Impact on Asthma guidelines recommend the use of second‐generation non‐sedating oral or intranasal H_1_‐antihistamines to treat the symptoms of AR, in combination with an intranasal corticosteroid or leukotriene receptor antagonist in cases where the symptoms are moderate/severe.^15^ AIT is then recommended in patients with moderate/severe AR and/or conjunctivitis who have a clinical history of symptoms despite use of symptom‐relieving medication and have a diagnosis of IgE‐mediated allergy.^15^

ITULAZAX® (SQ tree SLIT‐tablet) is a new sublingual immunotherapy (SLIT) tablet for the treatment of moderate‐to‐severe AR and/or conjunctivitis induced by pollen from the birch homologous group, indicated in adult patients with a clinical history of symptoms despite use of allergy pharmacotherapy and a positive test of sensitization to a member of the birch homologous group (skin prick test and/or specific IgE). The safety and efficacy of ITULAZAX® was demonstrated in the TT‐04 trial, a large‐scale, prospective Phase III, randomized, parallel‐group, double‐blind, placebo‐controlled multicenter study.^16^, ^17^ TT‐04 showed that ITULAZAX® resulted in a significant reduction in AR symptoms and medication use relative to placebo, with the total combined rhinoconjunctivitis symptom and medication score reducing by 39.6% during the birch pollen season (*p* < 0.0001).^16^, ^17^ Relative to subcutaneous immunotherapy (SCIT) products such as ALUTARD SQ® *Betula verrucosa* (ALUTARD SQ®), the sublingual administration of SLIT‐tablets offers several advantages, including at‐home administration, and increased convenience, especially for those living far from treatment facilities.^18^

A systematic review of cost data on AR in selected European countries (Denmark, France, Germany, Italy, and Sweden) concluded that there is a considerable economic burden associated with AR, driven mainly by indirect costs arising from high levels of absenteeism and presenteeism.^19^ Given this economic burden and the increasing pressure to optimize healthcare expenditure, the objective of the present study was to evaluate the relative costs of ITULAZAX® and ALUTARD SQ® *Betula verrucosa* in the treatment of moderate‐to‐severe AR from a societal perspective in Denmark.

## 2 METHODS

### 2.1 Cost‐minimization analysis and model

Cost‐minimization was selected as the most appropriate analysis methodology (as opposed to a cost‐effectiveness, cost‐benefit, or cost‐utility analysis) based on the assumption that ITULAZAX® and ALUTARD SQ® are clinically equivalent. While no head‐to‐head randomized trial data are available comparing ITULAZAX® with ALUTARD SQ®, a placebo‐controlled, randomized controlled trial (RCT) comparing the efficacy of SLIT and SCIT in the treatment of adults with birch pollen allergy showed no significant differences in rhinoconjunctivitis symptom scores or medication scores between SLIT and SCIT.^20^ The assumption of equivalence was further supported by analyses comparing SLIT and SCIT products in patients sensitized to other allergens. For instance, two meta‐analyses comparing SLIT‐tablets with SCIT in individuals with grass pollen allergy concluded that their efficacy was comparable.^21^, ^22^ Furthermore, a 2017 review by Brunton et al. reported that five small (*N* ≤ 71 patients) head‐to‐head, double‐blind, placebo‐controlled trials had directly compared the efficacy of SLIT with SCIT, including the aforementioned birch pollen allergy RCT, noting that “in the four trials that conducted statistical analyses, no significant differences in symptom scores were found between SCIT and SLIT”.^23^ It has also been demonstrated that, regardless of the administration route, AIT results in similar immunologic changes including increased IgG4‐levels.^24^ Previous economic comparisons of SCIT and SLIT‐tablets for individuals with allergies to other allergens have also been conducted as cost‐minimization analyses.^25^, ^26^

The cost‐minimization model was developed in Microsoft Excel 2016 to evaluate the costs associated with ITULAZAX® relative to ALUTARD SQ®, capturing all relevant treatment‐associated costs, persistence with treatment, and indirect costs arising from absenteeism and presenteeism. The analysis was conducted and written up in line with the Consolidated Health Economic Evaluation Reporting Standards checklist.^27^

### 2.2 Modeled patient population

In line with the approved indication for ITULAZAX® and the enrollment criteria for the TT‐04 phase III, randomized, double‐blind, placebo‐controlled, trial of ITULAZAX®, the population under consideration consisted of individuals 18–65 years of age with moderate‐to‐severe AR and/or conjunctivitis induced by pollen from the birch homologous group, with a clinical history of symptoms despite use of allergy pharmacotherapy and a positive response to a birch extract skin prick test (SPT); a positive IgE against Bet v 1 (the major allergen of birch pollen). The analysis focused on modeling costs in AIT‐naïve individuals initiating treatment with AIT, as opposed to individuals switching from an existing AIT. Population characteristics were derived from the TT‐04 trial, in which 47% of individuals were male, and the mean baseline age was 36.1 years (standard deviation 13.6 years). Note that the present study is based on clinical data from previously‐conducted studies and does not contain any data from studies of human or animal participants performed by any of the authors.

### 2.3 Perspective, time horizon, and discounting

The analysis compared the costs of ITULAZAX® with ALUTARD SQ® from a Danish societal perspective over a 3‐year time horizon, corresponding to the length of treatment to achieve disease modification recommended in international treatment guidelines. Future costs were not discounted in the base case analysis because the short time horizon and nature of the costs under consideration are comparable to those in a budget impact analysis in which discounting of future costs is not recommended.^28^ Discount rates were explored in one‐way sensitivity analyses.

### 2.4 Dosing and resource use

ITULAZAX® and ALUTARD SQ® dosing was modeled in line with the respective summaries of product characteristics (SmPCs). One ITULAZAX® tablet was assumed to be taken daily, while for ALUTARD SQ®, the “conventional initial therapy” up‐dosing scheme was used (Table S1), followed by a transition via one 100,000 SQ‐U injection after 2 weeks, one 100,000 SQ‐U after another 4 weeks, and then an ongoing 6‐weekly maintenance dose of 100,000 SQ‐U. Both treatments were assumed to be taken perennially in line with the SmPCs. As AIT has been shown to bring about a long‐lasting disease‐modifying effect, the European Academy of Allergy and Clinical Immunology Guidelines note that: “for patients with AR a minimum of 3 years of AIT is recommended to achieve long‐term efficacy after treatment discontinuation”.^29^ Based on treatment guidelines, all individuals using ITULAZAX® or ALUTARD SQ® incurred the cost of 3 years of treatment. In the base case analysis, adherence to both treatments was assumed to be 100%.

**FIGURE 1:**
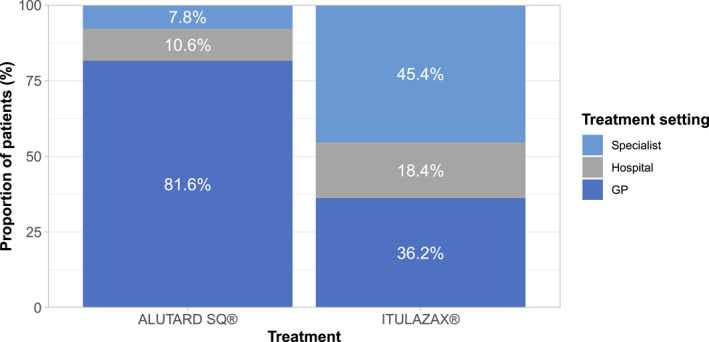
Distribution across healthcare settings during treatment with ALUTARD SQ® and ITULAZAX®

The base case analysis evaluated a scenario spanning three different care settings in Denmark: general practice, specialist, and hospital‐based care. The distribution between care settings during SCIT and SLIT treatment initiation and maintenance were based on 2018 data from the Danish Health and Medicines Authority (own calculations based on numbers from Sundhedsdatastyrelsen; Figure 1). The specialist tariffs were based on the current tariff values for an ear‐nose‐throat (ENT) specialist. The total number of visits was assumed to be the same across the three care settings for the given treatment modality (SCIT or SLIT); after a first dose visit, individuals treated with ITULAZAX® were assumed to require one annual evaluation consultation with a general practitioner (GP), specialist, or at the hospital depending on the care setting (Figure 1). Individuals treated with ALUTARD SQ® were assumed to require one GP appointment, specialist consultation, or hospital appointment for every injection. In year 1 (covering the up‐dosing phase; Table S1), this would correspond to a total of 22 healthcare professional (HCP) interactions: 15 titration visits, 2 transition visits, and 5 maintenance visits. This would then be followed by an average of 8.7 HCP interactions per year for administration of the ALUTARD SQ® maintenance dose in all subsequent years.

### 2.5 Costs

Costs of interacting with HCPs were based on a tariff‐based microcosting approach (i.e. based on the sum product of a tariff‐based unit cost and resource use estimate). Costs in each setting were calculated on a per‐visit basis based on tariff data (Table 1).^30^, ^31^, ^32^ In the specialist setting, the annual visit for SLIT, and one of the 8.7 visits for SCIT, were coded as the annual control visit which, in the case of SCIT, was assumed to cover administration of one of the maintenance injections.

**TABLE 1:** Costs of healthcare professional (HCP) interactions included the base case analysis

	Cost per consultation (DKK)	Reference
Allergy specialist tariffs		
ENT specialist first SLIT dose	478.76	Tariff card 11A 0144^28^
ENT specialist annual allergy control visit SLIT	555.72	Tariff card 11A 2027^28^
ENT specialist SCIT dose for patient with one allergy	485.43	Tariff card 11A 0143^28^
ENT specialist annual allergy control visit SCIT	555.72	Tariff card 11A 2026^28^
Hospital tariffs		
Hospital visit for uncomplicated SLIT or SCIT administration	2129	DRG 49PR03^29^
GP tariffs		
Consultation(s) subsequent to diagnosis	147.85	General practice tariff 0101^30^

Abbreviations: DKK, 2021 Danish Kroner; DRG, diagnosis‐related group; ENT, ear, nose, and throat; GP, general practitioner; SCIT, subcutaneous immunotherapy; SLIT, sublingual immunotherapy.

The list prices of ITULAZAX® and ALUTARD SQ® was obtained from the Danish Medicines Agency (Lægemiddelstyrelsen; medicinpriser.dk; Table 2).^33^ The cost of ALUTARD SQ® was based on the “Alk (108) *Betula verrucosa*” titration pack price during the up‐dosing phase, followed by the 100,000 SQ‐U/ml maintenance pack price for all injections after the up‐dosing. Sublingual immunotherapy costs were captured in the GP, allergy specialist, and hospital settings, while SCIT costs were only captured as a separate cost in the GP and allergy specialist settings as the 2021 hospital diagnosis‐related group tariff covers SCIT acquisition costs. Costs of pharmacotherapy to alleviate symptoms of allergy (such as nasal corticosteroids and antihistamines) were not captured based on the assumption that there would be no difference in the amount of pharmacotherapy used between patients treated with SCIT and SLIT.

**TABLE 2:** Allergy immunotherapy (AIT) acquisition costs in the base case analysis

	Price (DKK)	Pack size
ITULAZAX®	737.87	30 tablets
ALUTARD SQ® *Betula verrucosa* titration pack	2306.10	4 × 5 ml
ALUTARD SQ® *Betula verrucosa* maintenance pack	2022.50	1 × 5 ml

Abbreviation: DKK, 2021 Danish Kroner.

All costs were expressed in 2021 Danish Kroner (DKK), with results also presented in their Euro (EUR) equivalents based on the 365‐day average exchange rate obtained from the European Central Bank as at December 31, 2021 (1 DKK = 0.134342 EUR).^34^

### 2.6 Indirect costs

The ability to capture indirect costs was built into the model using a human capital approach based on absenteeism and presenteeism data from the TT‐04 trial (Table 3).^35^, ^36^, ^37^ The human capital approach captured the average wages of males and females, national unemployment rates, the average age of entry into the workforce, average age of retirement, and average number of days worked per year. It was assumed that the opening hours of healthcare facilities would align with the typical working week and that all HCP interactions for patients in the workforce would therefore take place during working hours. Rates of disease‐related absenteeism and reduced productivity levels (presenteeism) were excluded from the base case analysis based on the assumption that they would be equivalent in individuals treated with ALUTARD SQ® as in those treated with ITULAZAX®; however, this assumption was explored in sensitivity analysis. The base case did capture treatment‐related productivity loss, specifically that associated with HCP interactions, based on the assumption that 2 h of productivity loss would be incurred with each HCP interaction. The 2 h of productivity loss was selected in line with a published budget impact analysis of ALUTARD SQ® in Denmark and based on daily practice in Denmark, including an assumption of 10 km of travel per visit.^25^

**TABLE 3:** Parameters for the human capital‐based productivity loss calculations

Human capital approach model inputs	Value	Reference
Mean age (SD), years	36.1 (13.6)	TT‐04 trial^16^
Proportional male, %	47.0	TT‐04 trial^16^
Average annual male salary (DKK)	381,400*	http://www.statistikbanken.dk ^33^*
Average annual female salary (DKK)	296,000*	http://www.statistikbanken.dk ^33^*
Average age at entry into the workforce (years)	18	Assumption
Average age at retirement (years)	67	OECD^35^
Unemployment rate	3.3%	http://www.statistikbanken.dk ^34^
Number of working days per year (days)	220	Assumption

*Personal income by type of income, sex, age, population, price unit and unit. State Bank Denmark Table INDKP201.

Abbreviations: DKK, 2021 Danish Kroner; OECD, Organization for Economic Co‐operation and Development.

### 2.7 One‐way sensitivity analyses

A series of one‐way sensitivity analyses were conducted to evaluate the sensitivity of model outcomes to changes in individual input parameters. The model time horizon was changed to establish the effect of the analysis duration on model results. Similarly, the discount rate was varied to characterize the effect of placing different values on cost savings experienced in the future.

Two analyses were conducted around indirect costs; one in which disease‐related absenteeism and presenteeism were captured in both treatment arms on the basis of the values reported in the TT‐04 trial, and one in which all indirect costs were excluded from the analysis. In the analysis of disease‐related absenteeism and presenteeism, levels of absenteeism and presenteeism were based on data covering the tree pollen season (TPS), with a duration of 50 days in line with the TPS duration in the TT‐04 trial. In the trial, individuals on placebo recorded 1.29% days as sick days caused by allergy symptoms during the TPS. For individuals treated with ITULAZAX®, the proportion of sick days was reduced by 42% in the TPS versus placebo, corresponding to a relative risk of 0.58. Mean productivity with placebo in the TT‐04 trial was 89.3%, which increased by 3.4%‐points with ITULAZAX® over the TPS.

Two analyses were conducted to explore the effect of suboptimal adherence to treatment. The first was based on an analysis of 2429 patients receiving SLIT, and 2109 patients receiving SCIT in Germany, in which adherence rates were 81% and 83%, respectively, in those patients persisting with treatment until the end of a 3‐year treatment period.^38^ The second was based on a single center analysis of 330 patients treated with AIT between 2003 and 2011, in which 39.0% of patients treated with SLIT dropped out of treatment, compared with 32.4% of patients treated with SCIT, although the difference was not significant (*p* = 0.22).^39^

In both adherence analyses, AIT acquisition and HCP interaction costs were only incurred in the proportions of patients deemed adherent. Four analyses were then run in which the HCP resource use assumptions were modified, one analysis in each of the three care settings (GP, specialist, and hospital) in which 100% of patients were assumed to be treated in that setting and a final analysis in which the hospital‐based up‐dosing schedule was shortened to eight up‐dosing injections over 16 weeks, for a total of 12 injections in the first year of treatment rather than 22. Finally, one analysis was run to establish the contribution of symptom‐relieving medication on the absolute costs of SCIT and SLIT. The proportion of patients requiring symptom‐relieving medications (consisting of desloratadine, mometasone nasal spray, and olopatadine eye drops), and the average doses of each were based on data from the TT‐04 trial, while prices were obtained from the from the Danish Medicines Agency (Lægemiddelstyrelsen; Table 4) (Table 5).

**TABLE 4:** Proportions of patients requiring symptom‐relieving medication and average dosing during the tree pollen season (TPS) based on the TT‐04 randomized controlled trial (RCT), and Danish acquisition costs as used in sensitivity analyses

Symptom‐relieving medication	Proportion	Average dose	Pack price (DKK)	Pack size
Desloratadine tablets	66.8%	21.7 tablets	183.75	100 tablets (5 mg)
Olopatadine eyedrops	39.6%	38.0 drops	96.25	5 ml (1 mg/ml)
Mometasone nasal spray	47.9%	38.4 sprays	34.20	140 × 50 µg

Abbreviation: DKK, 2021 Danish Kroner.

**TABLE 5:** Cost‐minimization analysis results from the Danish base case analysis presented in Danish Kroner and converted to Euros using the 2021 365‐day average exchange rate from the European Central Bank

	ALUTARD SQ®	ITULAZAX®	Difference
Results in Danish Krone			
Total direct cost (DKK)	34,067	29,468	−4,600
Medication (DKK)	8,930	26,858	+17,928
Allergy specialist (DKK)	15,772	777	−14,995
Hospital (DKK)	8,910	1,564	−7,347
GP (DKK)	455	269	−187
Total indirect cost (DKK)	15,050	1,528	−13,522
Total cost (DKK)	49,117	30,996	−18,121
Results in Euros			
Total direct cost (EUR)	4,577	3,959	−618
Medication (EUR)	1,200	3,608	+2,408
Allergy specialist (EUR)	2,199	104	−2,014
Hospital (EUR)	1,197	210	−987
GP (EUR)	61	36	−25
Total indirect cost (EUR)	2,022	205	−1,817
Total cost (EUR)	6,598	4,164	−2,434

## 3 RESULTS

**FIGURE 2:**
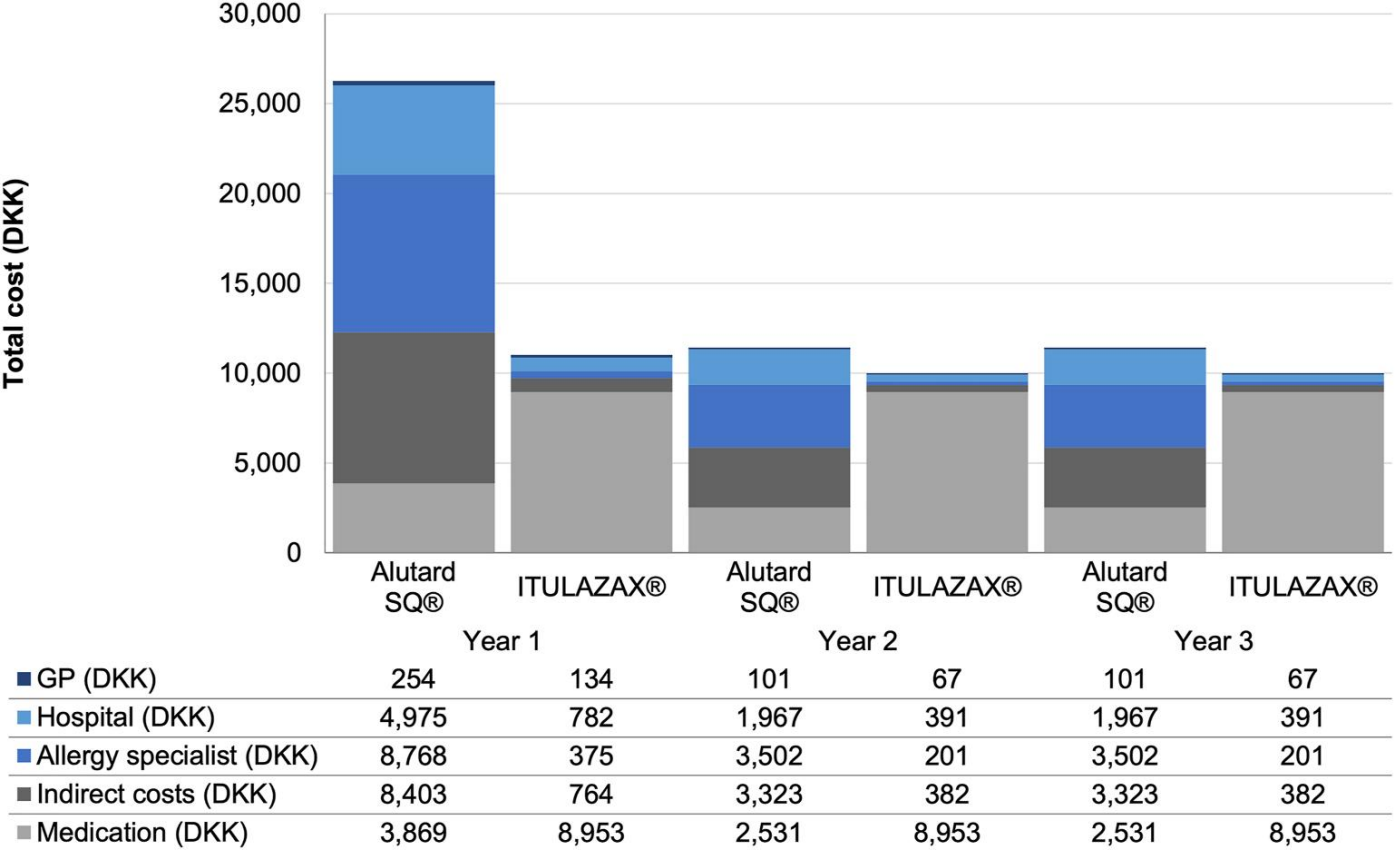
Base case analysis of annual per‐patient costs of allergy immunotherapy (AIT), healthcare professional (HCP) interactions, and indirect costs over the 3‐year analysis time horizon

The total cost of treatment over 3 years was estimated to be DKK 49,117 (EUR 6598) with ALUTARD SQ®, compared with DKK 30,996 (EUR 4164) in the scenario with ITULAZAX®, reflecting a cost‐saving of DKK 18,121 (EUR 2434; 36.7%) with ITULAZAX® over 3 years (Table 4). Over the 3‐year time horizon, AIT costs were predicted to increase by DKK 17,928 (EUR 2408), while costs of HCP interactions were predicted to decrease by DKK 22,528 (EUR 3026), more than offsetting the increased AIT costs (Table 4). The remainder of the cost savings associated with ITULAZAX® arose from reductions in indirect costs. The annual direct costs of AIT and HCP interactions and all indirect costs are shown over the model time horizon in Figure 2.

One‐way sensitivity analyses showed that incremental model outcomes in the base case were driven primarily by cost savings in the hospital setting. Aside from differences in the costs in each care setting, the model was broadly insensitive to changes in individual input parameters, such as the discount rate or the choice of ALUTARD SQ® up‐dosing scheme. One notable exception was the exclusion of the 2 h of lost productivity costs associated with each HCP interaction, which decreased the cost savings with ITULAZAX® from DKK 18,121 (EUR 2434) in the base case to DKK 4600 (EUR 618) in the analysis excluding productivity loss differences arising from HCP interactions (Table 6). Changes to assumptions around the care setting showed that ITULAZAX® would be cost‐saving to greatly varying degrees in the hospital, specialist, and GP settings, saving DKK 72,022 (EUR 9676; 66.1%), DKK 13,846 (EUR 1860; 31.2%), and DKK 1889 (EUR 254; 6.2%) respectively. Changing the rates of adherence to treatment had a substantial effect on the absolute cost estimates, but only reduced the cost savings with ITULAZAX® by EUR 192 in the analysis based on Allam et al. and increased the cost savings with ITULAZAX® by EUR 455 in the analysis based on Lemberg et al.

**TABLE 6:** One‐way sensitivity analysis results presented in Danish Kroner and converted to Euros using the 2021 365‐day average exchange rate from the European Central Bank

Analysis	ALUTARD SQ®	ITULAZAX®	Difference
Base case	DKK 49,117	DKK 30,996	DKK‐18,121
	*EUR 6598*	*EUR 4146*	*EUR‐2434*
3% discount rate	DKK 48,129	DKK 30,273	DKK‐17,855
	*EUR 6466*	*EUR 4067*	*EUR‐2399*
5% discount rate	DKK 47,511	DKK 29,822	DKK‐17,689
	*EUR 6383*	*EUR 4006*	*EUR‐2376*
No indirect costs	DKK 34,067	DKK 29,468	DKK‐4600
	*EUR 4577*	*EUR 3959*	*EUR‐618*
Include disease‐related absenteeism and presenteeism from TT‐04 trial (equal in both arms)	DKK 65,669	DKK 47,547	DKK‐18,121
	*EUR 8822*	*EUR 6388*	*EUR‐2434*
Adherence in line with Allam et al.	DKK 43,326	DKK 25,397	DKK‐17,929
	*EUR 5821*	*EUR 3412*	*EUR‐2409*
Adherence in line with Lemberg et al.	DKK 38,079	DKK 19,503	DKK‐18,576
	*EUR 5116*	*EUR 2620*	*EUR‐2496*
All hospital‐treated patients treated with accelerated ALUTARD SQ® up‐dosing	DKK 36,754	DKK 30,868	DKK‐5887
	*EUR 4938*	*EUR 4147*	*EUR‐791*
All patients treated by a GP	DKK 30,867	DKK 28,978	DKK‐1889
	*EUR 4147*	*EUR 3893*	*EUR‐254*
All patients treated in the hospital setting	DKK 108,924	DKK 36,902	DKK‐72,022
	*EUR 14,633*	*EUR 4957*	*EUR‐9676*
All patients treated by an ENT specialist	DKK 44,378	DKK 30,532	DKK‐13,846
	*EUR 5962*	*EUR 4102*	*EUR‐1860*
All treatment initiations in the ENT specialist setting, followed by the base case split between care settings	DKK 46,903	DKK 30,868	DKK‐16,035
	*EUR 6301*	*EUR 4147*	*EUR‐2154*
Including symptom‐relieving medication costs	DKK 49,254	DKK 31,132	DKK‐18,121
	*EUR 6617*	*EUR 4182*	*EUR‐2434*

Abbreviations: DKK, 2021 Danish Kroner; ENT, ear, nose and throat; EUR, 2021 Euros; GP, general practitioner; SCIT, subcutaneous immunotherapy.

## 4 DISCUSSION

Based on a short‐term (3 years) cost‐minimization analysis, treatment with ITULAZAX® would save DKK 18,121 (EUR 2434) per patient relative to ALUTARD SQ® from a societal perspective in Denmark. The acquisition costs of ITULAZAX® were higher than those of ALUTARD SQ®, but the higher costs were offset by reduced costs associated with HCP interactions. Beyond the cost differences between healthcare settings, sensitivity analyses showed the model to be most sensitive to the assumption of 2 h of lost productivity associated with HCP interactions, with cost savings decreasing to DKK 4600 (EUR 618) when this cost was omitted from the analysis. Other sensitivity analyses showed the model to be relatively insensitive to changes in model input parameters, such as discount rate and adherence. The absolute and incremental costs varied considerably between the three different care settings, with ALUTARD SQ® treatment costing DKK 30,867 (EUR 4147) over 3 years in the GP setting and DKK 108,924 (EUR 14,633) in the hospital setting, and ITULAZAX® treatment costing DKK 28,978 (EUR 3893) in the GP setting and DKK 36,902 (EUR 4957) in the hospital setting. Focusing exclusively on the hospital setting, the difference in costs relative to ALUTARD SQ® was shown to narrow with the use of an accelerated up‐dosing schedule, illustrating the substantial contribution of SCIT up‐dosing to overall costs.

The present study has limitations that should be acknowledged when interpreting the findings. The first potential limitation is the evidence base on which clinical equivalence was assumed. As no head‐to‐head clinical data are available comparing ITULAZAX® with ALUTARD SQ® directly, the assumption was based on meta‐analyses comparing SCIT and SLIT products for treating allergies to other allergens, and immunological data on changes in IgG4 levels regardless of AIT administration route. There is also precedent for utilizing the cost‐minimization analysis to compare SCIT with SLIT for other allergens. The absence of evidence of a difference does not, however, prove that there is no difference between treatments.^40^ Indeed, it is not possible to definitively demonstrate that two treatments have an identical effect.^41^ Based on a combination of clinical data on ITULAZAX® from TT‐04 and meta‐analyses comparing other SCIT and SLIT products, it is unlikely that any differences in efficacy, should they exist, would be clinically meaningful.^21^, ^22^ One area in which differences may exist between SCIT and SLIT is in the incidence and nature of local adverse reactions. Between 26% and 86% of patients experience local reactions to SCIT such as pruritus and/or erythema (>2.5 cm), while the most common reactions to SLIT involve the oromucosal or gastrointestinal regions.^42^ However, reactions tend to be mild in nature and occur most frequently during the titration phase of treatment and would therefore be unlikely to drive meaningful differences in incremental economic outcomes.^40^

Given the assumption of clinical equivalence, no further differences in cost outcomes would be anticipated between the treatments beyond those reported, but additional absolute direct and/or indirect costs may be incurred in patients with AR. For instance, the Bet v 1 allergen is cross‐reactive with the major allergens of certain foods, which can result in individuals experiencing pollen‐food syndrome (PFS). Characteristic symptoms of PFS include itching of the lips, tongue, and throat, sometimes accompanied by swelling.^1^ A 2011 study found PFS to be highly prevalent in patients with birch‐related AR, with 73.3% of individuals experiencing symptoms associated with eating certain types of food, 86% of whom experienced PFS perennially.^43^ Secondly, the costs of asthma were not captured in the present analysis. While between 15% and 38% of patients with AR have allergic asthma, given the assumption of equivalence between SCIT and SLIT, the exclusion of asthma treatment costs would not be anticipated to drive a further difference in costs between SCIT and SLIT.^15^, ^44^, ^45^

In the base case analysis, adherence to both treatments was assumed to be 100% to facilitate an unbiased comparison, increase the interpretability of the findings, and comply with the foundational assumption of equivalent efficacy in cost‐minimization analyses. Evidence from real‐world studies suggests that patient adherence to SLIT and SCIT products is lower, with previous studies reporting adherence rates to SCIT and SLIT products to be highly variable; between 7% and 81% for SLIT and between 23% and 83% with SCIT.^37^, ^46^ Sensitivity analyses were conducted to evaluate the effect of non‐adherence in the present analysis based on the assumption that non‐adherent patients would not fill their AIT prescriptions and, in the case of SCIT, not incur the cost of an HCP interaction for the subcutaneous injection.

Finally, the Danish Agency for Patient Complaints recently handled a case (case number 17/1238/KC) in which a complaint was raised noting that subcutaneous AIT had to be bought at the pharmacy while other hospital medications were generally provided by the hospital without any direct cost to the patient. The Agency ruled that the current practice conflicted with Danish law, with the verdict mandating that all Danish hospitals need to provide and cover the costs for subcutaneous AIT. This is distinct from SLIT prescribed in the hospital setting, where there is a co‐payment for the SLIT cost, and also from the GP and specialist settings, where there is a co‐payment for both SCIT and SLIT costs. The decision has resulted in a substantial incentivization for patients to continue to receive SCIT therapy in the hospital setting rather than transitioning into general practice, which has in turn resulted in disparities in access to SCIT, particularly in rural areas of Denmark. The present analysis may be affected by these decisions, most notably in shifting the balance between care settings for SLIT and SCIT, and indeed potentially suppressing uptake of SLIT in Denmark, despite the projected cost savings relative to SCIT.

## 5 CONCLUSION

The present analysis showed that ITULAZAX® would result in cost savings relative to ALUTARD SQ® from a Danish societal perspective, and ITULAZAX® would therefore be a clinically and economically prudent alternative to ALUTARD SQ® in Denmark for the treatment of patients 18–65 years of age with moderate‐to‐severe AR and/or conjunctivitis induced by pollen from the birch homologous group with a clinical history of symptoms despite use of allergy pharmacotherapy.

## AUTHOR CONTRIBUTION

**Steen M. Ronborg**: Methodology; Supporting, Supervision; Supporting, Writing – review & editing; Lead. **Tobias Sydendal Grand**: Conceptualization; Supporting, Funding acquisition; Supporting, Project administration; Supporting, Writing – review & editing; Supporting. **Henrik Brandi**: Conceptualization; Supporting, Funding acquisition; Lead, Project administration; Lead, Supervision; Lead, Writing – review & editing; Lead. **Richard F. Pollock**: Conceptualization; Lead, Formal analysis; Lead, Methodology; Lead, Project administration; Supporting, Validation; Lead, Visualization; Lead, Writing – original draft; Lead, Writing – review & editing; Supporting.

## CONFLICTS OF INTEREST

Steen M. Ronborg has received advisor and consultancy fees from ALK‐Abelló A/S; Henrik Brandi and Tobias Sydendal Grand were full‐time employees of ALK‐Abelló A/S at the time of the study; and Richard F. Pollock is the director and shareholder of Covalence Research Ltd, which received consultancy fees from ALK‐Abelló A/S to conduct the analyses and draft the manuscript.

## Supporting information

Supporting Information S1
