# Supplementary material for: ITULAZAX® versus Alutard SQ® in the treatment of allergic rhinitis induced by pollen from the birch homologous group: A cost‐minimization modeling analysis from the Danish societal perspective
Source: Clin Transl Allergy. 2022 Nov 3;12(11):e12196. doi: 10.1002/clt2.12196 (PMC9631325; doi:10.1002/clt2.12196)
Supplement: Supplementary file 1 — Supporting Information S1 [file CLT2-12-e12196-s001.docx]

| Injection number/week | Vial | Injection volume (mL) | Administered dose SQ-U/injection |
| --- | --- | --- | --- |
| 1 | 1 (100 SQ-U/mL) | 0.2 | 20 |
| 2 |  | 0.4 | 40 |
| 3 |  | 0.8 | 80 |
| 4 | 2 (1,000 SQ-U/mL) | 0.2 | 200 |
| 5 |  | 0.4 | 400 |
| 6 |  | 0.8 | 800 |
| 7 | 3 (10,000 SQ-U/mL) | 0.2 | 2,000 |
| 8 |  | 0.4 | 4,000 |
| 9 |  | 0.8 | 8,000 |
| 10 | 4 (100,000 SQ-U/mL) | 0.1 | 10,000 |
| 11 |  | 0.2 | 20,000 |
| 12 |  | 0.4 | 40,000 |
| 13 |  | 0.6 | 60,000 |
| 14 |  | 0.8 | 80,000 |
| 15 |  | 1.0 | 100,000 |
